# Self-management guidelines for youth who have lost a family provider through HIV/AIDS

**DOI:** 10.4102/hsag.v28i0.2171

**Published:** 2023-08-28

**Authors:** Siphesihle D. Hlophe, Karien Jooste

**Affiliations:** 1 Department of Nursing Science, Faculty of Health and Wellness Sciences, Cape Peninsula University of Technology, Cape Town, South Africa

**Keywords:** HIV/AIDS, self-management, family, CHC, youths, emotional, financial

## Abstract

**Background:**

When parents die from HIV/AIDS-related causes, children often experience emotional instability and are given additional obligations, such as caring for siblings. Youths may react in a variety of ways, including increasing alcohol consumption, and their relationships with their siblings may be altered positively or negatively.

**Aim:**

The purpose of this article is to examine the lived experiences of youths in managing themselves after losing a family member to HIV/AIDS and suggest developed guidelines for nurses to advise youths on self-management after losing a family member to HIV/AIDS.

**Setting:**

Khayelitsha, Western Cape province, South Africa.

**Method:**

A descriptive phenomenological design for this study was followed. The researcher conducted 11 semi-structured interviews with participants. The study was conducted with participants that were youth aged between 18 and 25 years.

**Results:**

The study revealed that the death of a family provider can be difficult for the youth left behind to deal with the changes in their daily lives.

**Conclusion:**

The findings demonstrated that the death of a family member has a significant impact on the family. One of the more senior family members must assume charge and remain strong to help their siblings focus on the future. The death of a family member might result in a cascade of forced changes that necessitate new behaviours to maintain stability.

**Contribution:**

This study’s context-based data focuses on how the Community Health Centre (CHC) may assist young people in managing themselves after a family provider has died from HIV/AIDS, using the developed guidelines.

## Introduction and background

As of 2015, it was projected that 13.4 million children and adolescents all over the world had lost either one or both of their parents. More than 80% (10.9m) of these youngsters are located in sub-Saharan Africa. In some nations that have been hit particularly hard by the pandemic, a significant proportion of all orphaned children are victims of HIV/AIDS, for instance, 74% of orphaned children in Zimbabwe and 63% of orphaned children in South Africa (AVERT 2017).

According to Li, Liang, Lin, Lan, Ji and Xiao (2019:1) approximately 13.4 million children world-wide are under the age of 18 years and have lost one or both parents to AIDS while millions more are living with HIV-positive parents or primary caregivers. According to UNAIDS (2018), 16.5m children around the world have lost either one or both of their parents to AIDS.

A family is frequently a source of emotional support, love, stability and protection, and it may give a distinctive feeling of belonging and values that cannot be obtained in other types of relationships. The advantages of having a family are numerous and widespread, and their reach is extensive (Revilla 2019). As a result, following the loss of a member of the family, the surviving members of the family will experience difficulties and health-related concerns, such as an inability to sleep, particularly in the first year following the tragedy (Glatt 2018:103). The remaining members of a family are no longer provided with the same level of support after the death of a family member. Parenting becomes a far more challenging endeavour for the surviving spouse (Glatt 2018:103). This may need the practice of self-management, which is nothing more than the act of engaging in deliberate self-care with the goal of assisting members of the family in feeling stronger, restoring their sense of serenity and providing them with hope for what lies ahead (Wolf 2019).

The degree to which people are able or willing to take charge of their own life and adapt to shifting circumstances is referred to as self-management (Lenzen et al. 2017:1). The increased autonomy and well-being are two benefits of practising effective self-management (Crowley et al. 2019). Self-management could help people cope with stressful events like losing a family member. Individuals who engage in self-management have better health outcomes, according to empirical data (Crowley et al. 2019) perplexed (Crowley et al. 2019). Self-management requires condition-specific knowledge, beliefs and self-regulation abilities (Crowley et al. 2019). Stress is inevitable, yet it can leave you feeling unfocused, overwhelmed and powerless. Find meaning in the struggle or situation you are currently in by making means to ensure that you receive adequate rest, stay active on a regular basis and make healthy food choices (Griffin 2019).

When a parent dies, children may feel worried, perplexity, behavioural changes or emotional withdrawal (Glatt 2018:114). Youths who lose their parents may become alcoholics and have different sibling relationships (Glatt 2018:115). Youths are still under parental supervision and either in school or employed. Youths are 18 to 25 years, as well as youthful, vivacious and immature (United Nations Educational, Scientific and Cultural Organization [UNESCO] 2017). Youths are emotionally unstable when a family member dies.

When a family member passes away, those left behind face numerous obstacles, worsening if the dead were a breadwinner. Participants’ testimonies demonstrated that they had to cope with emotional and practical loss implications. To relieve stress, many people turned to hazardous behaviour like drinking and smoking, according to the research.

The research study that was done took a qualitative approach. The interviews were semi-structured with 11 participants. These participants were young individuals who had lost a family member to HIV/AIDS. Most young women interviewed in this study showed shock and disbelief when they realised a close one had died for a mysterious reason. These reasons include not disclosing HIV/AIDS status to the family members, not seeking medical attention after their diagnosis of HIV/AIDS and defaulting on HIV/AIDS medication after they had started; this caused anger, remorse and guilt. Despite their financial needs and family tensions, bereaved relatives struggle to stay stable and see a future. A few survey participants recalled visiting neighbourhood self-help groups, seeing a social worker at the local clinic or chatting with someone at church. According to their comments from interviews that took place in January and February 2020, many participants knew they needed therapy and felt it would help.

This article is based on a larger study, the Master of Nursing, where the researcher published a thesis titled ‘Guidelines for nurses at a comprehensive primary healthcare clinic in Cape Town, with which to advise youths on self-management following the loss of a family member with HIV/AIDS’ for the purposes of obtaining the qualification.

## Problem statement

In order to improve the process of designing and delivering self-management interventions, it is important to have a comprehensive understanding of the individual experiences that result in pain (Devan et al. 2018:382). After the passing of a family provider who battled HIV/AIDS, young people may go through a range of emotions, including anxiety and depression. Due to HIV/AIDS, some families are forced to transition into a situation where a younger member of the family must take on the role of becoming a family provider (Glatt 2018:107). Taking up such a big role at a young age may lead to physical discomfort where they experience a significantly increased risk of clinical depression. The result of such experience results in youth being less likely to participate in active self-management measures (Devan et al. 2018:394).

Most youths attend public clinics because of panic attacks and anxiety following a family member’s death (Devan et al. 2018:394).

Research questions included the following:

- *What are the lived experiences of youth who have lost a family provider through HIV/AIDS?*
- *What guidelines are in place at the Community Health Centre (CHC) to support the youths on how to manage themselves after losing a family member to HIV/AIDS?*

## Research purpose

The purpose of the research were to:

- Examine the lived experiences of youths in managing themselves after losing a family member to HIV/AIDS.
- Develop guidelines for nurses to advise youths on self-management after losing a family member to HIV/AIDS.

## Method

This research took a qualitative approach. Non-probability sampling was followed in this qualitative research as the researcher was unable to locate the entire population (Brink, Van der Walt & Van Rensburg 2018:124; Hlophe 2020; 24). Non-probability sampling is constructed from an objective judgement as a starting point, and the direction the sampling takes will be a decision made by the researcher as the study progresses (Gray et al. 2016). Purposive sampling was based on the researcher’s judgement regarding participants who are typical of or especially knowledgeable about the study phenomenon. Participants were selected based on the features or characteristics which will help to explore the research questions (Brink et al., 2018:159; Hlophe & Jooste 2022:2). This research used semi-structured interviews with 11 young individuals who had lost a family member to HIV/AIDS.

The data collection method was phenomenological (Ellis 2019). A phenomenological approach to interviews allows the researcher to obtain a more profound knowledge of a phenomenon by asking probing questions that provide information about the individual’s lived experience (Ellis 2019).

### Data generation

A multilingual researcher translated Xhosa transcripts into English. Transcribing seemed simple, but it required decisions concerning detail level, data interpretation and presentation. Data analysis began with attentive listening and observing to transcribe data (Daniel 2016).

After the interviews, all data and field notes were coded. The researcher translated local interviews into English, and then an editor who speaks both languages back translated them.

Qualitative data analysis reduces narrative non-numerical data to themes and categories using a coding technique in qualitative data analysis (Brink et al. 2018:46). Thus, ATLAS.ti version 8 categorised and themed data. An independent programmer verified open coding. ATLAS.ti version 9 programme was used in qualitative research and data processing.

The researcher and the unit manager of the CHC were in communication. The unit manager of the CHC in Cape Town was called to place posters asking clients to volunteer after their check-ups or appointment. The study’s purpose and details were posted. The unit manager granted permission to use a private room for studies. The unit manager was told the study’s purpose and the advantages of teaching youngsters how to cope with HIV/AIDS loss. Before conducting semi-structured interviews, the researcher told the board of directors about the study’s aim. The researcher said he would have HIV/AIDS and other health conversations with patients. The research participants were 18–25 years.

### Study recruitment flow

Between January 2020 and February 2020, the researcher would explain the purpose of the study every day in the morning to the nursing staff working in the consultation room and treating patients. The primary purpose was to request their assistance in determining the possible candidate for the study and refer them to the researcher. The possible participants had to be willing to participate in the study voluntarily (Hlophe & Jooste 2022:3). Following consultations with patients, those who agree to see the researcher and where possible potential participants from the study were requested to go see the researcher in the private room for the interviews. The researcher would receive the willing and potential participants and explain the purpose of the study to each individual, should they be still willing to participate they would, and he would proceed to provide an information letter and obtain informed consent. The posters also played a role in the recruitments as some of the participants came to the researcher without being recruited by the nursing staff as they were not at the CHC for consultation purposes (Hlophe 2020:24). The participants were then informed about the study’s goal. Their involvement is voluntary, and they may quit at any time. Participants might have been interviewed at home, but they all chose a private clinic room. The interviews were conducted without disturbances in a private room. Everyone felt comfortable during semi-structured interviews, and those who felt emotional strain from the interviews were referred to the social worker who was present at the CHC for further management. Each participant who felt emotional was asked if they could continue with the interview and all allowed the research to continue. Finally, participants signed informed consent (Hlophe 2020:24).

The participants were 23 years old on average, with ages ranging from 19 to 25 years. All participants had a family member who had died of HIV/AIDS. Six were in school or college, and five were working. Two participants’ moms died, while nine others lost relatives.

### Data analyses

The researcher converted specific isiXhosa transcripts into English. Some of the participants were illiterate and found it more helpful to answer the questions in their home language.

After analysis, some of the information was classified into two categories, but quotes were categorised into the topic and type that best expressed the subject. After recognising meaning units, the researcher reread the original text with a list of meaning units to determine whether all context important to the study’s objective has been addressed. The categorisation is the third phase. Units were decreased without losing their content before the extended meanings. Then, after the categories have been determined, the researcher’s analysis and writing up process began.

After that, the field notes and interview data were coded. All were English interviews, but few participants had occasions where they felt it was best to express themselves in the isiXhosa language. The interviewer had the responsibility to translate isiXhosa into English. An editor who was fluent in both languages ensured the accuracy of the translation.

Data analysis from the 11 interviews resulted in the following four major themes (Box 1):

BOX 1 Overall themes of the study.

**Table:** 

**Themes**
Time-related circumstances define behaviour to manage the death of a family member Youth go through different stages after the unexpected loss of a family member Managing difficult changes in the daily lives of the next of kin Support measures for the next of kin

*Source:* Hlophe, S.D., 2020, ‘Guidelines for nurses at a comprehensive primary healthcare clinic in Cape Town, with which to advise youths on self-management following the loss of a family member with HIV/AIDS’, Doctoral dissertation, Cape Peninsula University of Technology

**Theme 1:** Time-related circumstances define behaviour to manage the death of a family member.

**Theme 2:** Youths go through different stages after the unexpected loss of a family member.

**Theme 3:** Managing complex changes in the daily lives of the next of kin.

**Theme 4:** Support measures for the next of kin (Hlophe & Jooste 2022: 6). This article is based on theme 3, which seeks to address how the CHC can support or advise the youths on how to manage themselves after losing a family member to HIV/AIDS.

An outside coder was hired to confirm accurate coding and that his version matched the researchers’. Although a coder’s primary purpose is to maintain measurement consistency, a coding helper was more suited due to the need for reliability. Independent coder reliability occurs when two or more independent coders have the same opinion on the coding of relevant content while using the same coding scheme (ed. Palazzo 2019).

### Ethical considerations

Ethical clearance to conduct this study was obtained from the Cape Peninsula University of Technology, Research and Ethics Committee of the Faculty of Health and Wellness (No. CPUT/HW-REC 2019/H2), as well as the Western Cape Province Department of Health (No: WC 201911 032).

## Results

Grieving family members may not fully understand the phases of their grief. Each step is time-related and necessary for the next (Lenzen et al. 2017). Grieving people frequently need others’ help. Mourners embrace change. Changes in daily life and habits generate adjustment and disturbance that may be difficult to handle. This, too, needs help. Individuals’ health may improve if they set self-management goals and strategies to promote self-efficacy and modify behaviours (Lenzen et al. 2017).

### Theme 3: Managing complex changes in the daily lives of the next of kin

In this theme, the problematic changes in the daily lives of youth after the deceased family member will be discussed. They must quickly move from family member to home authority and family earner and cope with financial requirements, family disagreements and community shame.

### Category: A rapid shift from family member to household authority and breadwinner

Due to the loss of a breadwinner and single parenting, children must care for their families (Adeojo 2017:45).

When a parent dies, someone needs to take care of the responsibilities that were for the parents in the household. The family’s older children wind up taking on tasks for which they need to prepare, and they frequently have to drop out of school (Shah 2018:8).

Grandma’s death deprived a participant of emotional support. She had no one to talk to about the changes at home:

‘And she was the one who was like a breadwinner because we depended on her [*stutters*], on – on her grant. Like it was tough because ever since she died, I never got the support that I used to get. Many things changed at home because there was no longer that person who used to support us and discuss life, about HIV and so on.’ [*Shakes while speaking, rubs nose*] (P5, 19 years, Female, Matric learner)

In many situations in South Africa, parents die before their children reach adulthood, leaving their offspring to grow up in extremely difficult circumstances, aggravated by poverty. Following the death of a parent, people’s daily lives and lifestyles alter (Apelian & Nesteruk 2017:80). Whether the abandoning was deliberate or not, a child may feel abandoned. Mkhize (2016) defines abandonment as not returning to a known person. This research shows that death might seem like abandonment.

In this research, behavioural techniques should include self-management support to help patients feel better.

Many participants had to let go of everything and hunt for work after the family’s breadwinner died:

‘It was so difficult for me because I was the older person in the house, so I had to struggle to go and look for a job.’ (P2, 25 years old, Female, College student)

‘As I said … uh … after my mom passed on, I had to take over as a breadwinner. So I’m currently, I am not full-time working. I am working part-time.’ (P1, 24 years old, Female, Pharmacist Assistance)

The death of parents or caregivers disrupts the stability of their children’s life:

‘And also, being the eldest of my siblings, my whole life had to change because now I have to become the breadwinner and support my younger siblings.’ (P1, 24 years old, Female, Pharmacist Assistance)

Youngsters of all ages are affected by parental death, but adolescent or elder siblings are particularly vulnerable. According to studies, their death disrupts the stability of their life and causes financial, social and emotional disturbances. After a mother’s death, household expenditures rise, and younger siblings may require help (Apelian & Nesteruk 2017:81). They must serve as role models to replace the void left by the deceased parent. Being a role model entails taking on a role that is passed down to the next participant after her sister’s death, which she may not be prepared for and demonstrating virtues like determination, hope, integrity, optimism and compassion. Positive child development necessitates using role models (Prince-Mitchell 2017).

When the family’s breadwinner died, the remaining family members faced numerous challenges, according to one participant:

‘She was the only one taking care of us, me and my siblings and my son. That is why we faced difficulties [*voice shakes, eyes fill with tears, tight body posture*].’ (P2, 25 years old, Female, College student)

Her responsibilities had to be perfect. Her father’s grant supported her and her siblings:

‘But now, I benefit from my dad’s grant. And that is why I wouldn’t say I like it. She always cared for me and about things and her children, but now it’s my responsibility to take care of them. It’s my responsibility now.’ (P4, 24 years old, Female, Studying grade 11)

Young adults and teenagers who are supporting themselves are responding to the current social and economic climate.

According to Apelian and Nesteruk (2018:233), girls are most affected by a mother’s death since they must become caretakers at a young age.

### Category: Financial needs

Food insecurity is a problem that may affect families of all economic levels, which may harm health following a death. Food insecurity is having little option about what to eat or not eating at all, as well as shame at being deemed poor (Morris, Fletcher & Goldstein 2019:2).

A participant reported they had nothing to eat when the breadwinner died and applied for a grant. To assist her siblings, the participant now runs a small business:

‘We had nothing to eat, neh. Now since we do have that grant and I do sell the small chips and sweets and stuff so that we can be able to buy bread or something short at home … she was the only one that was working at home; she was the only one that was putting food on the table [*strokes chin*].’ (P2, 25 years old, Female, College student)

Stack and Meredith (2018:233) found that when families are low on money, they often have to make compromises on food, making it harder to meet the nutritional needs of their children. The people who participated in this study said that they were hungry and struggling to make ends meet.

A participant talked about how her family-run business helped her provide for her little child and her siblings:

‘And I did find the money to start a small business for me so that I can put something on the table for my aunt’s kids and my baby as well [*looks very sad*].’ (P2, 25 years old, Female, College student)

P2’s aunt’s death has strained her finances:

‘No one working at home unless it was only my aunt working, right? Now for that fact that she had died of HIV. Now things were getting hard at home; we had nothing to eat and … uh … there was no one to look after us.’ (P2, 25 years old, Female, College student)

Many people are drawn into financial debt due to the death of a family member, according to Adams (2020).

Individuals’ mourning is prolonged when they face financial challenges due to a death. Economic hardship caused by a loved one’s death leads to low life satisfaction (Morris et al. 2019:332).

### Category: Family fights and not dealing with reality

According to Feigelman et al., young widows are often subjected to familial violence and conflict (2017:138). Death of a parent or family member may cause delinquency, depression, suicidal thoughts and drug abuse (Feigelman et al. 2017:136). These activities exacerbate family feuds.

Rice and Tan (2017:82) looked at patients who were hospitalised for psychiatric reasons after the death of a family member. The causes of their psychological problems were found to be no communication with parents(s), physical and verbal fights with parents or siblings, parental conflicts and a lack of parental involvement.

A participant’s sister buried her sentiments since she was not emotionally open before her sister died. She stated:

‘Fights. Family fights. She [*her sister*] doesn’t want to talk. She’s always quiet. She keeps things inside of her. And when she hurts, she doesn’t want to talk about that thing she hurt about [*looks exhausted and throws hands in the air*].’ (P4, 24 years old, Female, Studying grade 11)

In an unsupportive environment, family members had to deal with the death of a loved one (Haravuori, Suomalainen & Marttunen 2016).

The prior participant missed her sister and told her about her sentiments and anguish:

‘When she [*her sister*] wants to talk, she calls me and talks to me and says, “This, I’m hurt, and I don’t like to talk too much, but I know it’s painful for me.” Now she’s gone, and we can’t do anything. Ja, she’s gone, and I miss her so much.’ (P4, 24 years old, Female, Studying grade 11)

A woman said disobedient children caused her strain. She did not want to say something she would later come to regret, so she stayed silent:

‘Yho, stress is a lot. Yho … I have lots of stress. [*Holds head and pulls hair*.] I’m stressed because her children are annoying me. [*Makes an angry face*.] When I’m saying someone must do that, they should do that. I always want to encourage children to listen because they make someone stressed all the time, to feel like … hmm, I feel like I should keep quiet sometimes so that that thing inside will make me regret what I am going to say.’ (P4, 24 years old, Female, Studying grade 11)

Death and suffering may produce unreasonable behaviour and destabilise a family. One reason for the difficulty in communicating after a death in the family is that people grieve in different ways (Haley 2020). Some family members may negotiate over their new obligations when new authority and behaviour patterns emerge. People suffer and react differently to changes in other family members’ behaviour (Haley 2015):

‘She [*the aunt*] was calling me a stupid girl because while my sister was alive, she said I take her for granted. And now that my sister is passed away, I want her to look after me, and she doesn’t have the money to look after me because she also has children [*body shakes while sharing*]. Where must I stay now, and whom do you stay with?’ (P9, 21 years old, Female, College student)

Krull (2020) argues that loss and self-centred loved ones might have negative effects. Family members who have just lost a loved one may encounter ‘narrow-minded relatives’, says Krull (2020). People do not always mourn healthily, and their sorrow may be destructive to themselves and others.

This suggests that teenagers should divert their attention away from unpleasant events.

After her sister died, a participant’s sister’s husband forced her out of the home, leaving her to live with her grandmother:

‘It was tough because I was staying with her and her husband and her three children. So, after she passed away, the husband chased us away. And we had to move back and stay with my grandmother [*sad face and shaking*].’ (P11, 25, Female, Voluntary work)

According to Levey et al., children who are placed in the care of others after the loss of a parent are at risk of neglect, exploitation and poor treatment (2017:10).

### Category: Stigma in the community

HIV/AIDS stigma affects children’s mental health. Peers devalue, victimise and reject them (Yassin, Erasmus & Frantz 2020). Bullying, discrimination, social isolation and the denial of education are just a few of the ways in which stigma affects children, adolescents and adults. Young people, teenagers and children who have lost a parent or parents and are living with extended family members frequently describe being abused and treated harshly (Yassin et al. 2020). As a result, it is critical to treat it with the concept that one should develop confidence in controlling one’s position.

Following the death of her mother, a participant described how she was stigmatised in the community:

‘And also, the stigma from my community is one of the things that I experienced after the loss of my mom [*emotional and sad facial expression*].’ (P1, 24 years old, Female, Pharmacist Assistance)

Children of parents living with or who died from HIV/AIDS are at a higher risk of developing poor self-esteem and depressive symptoms than their peers (Domlyn et al. 2020).

People in the community talked behind her back because of the stigma, according to one participant:

‘So basically, what happened after my mom passed was most of the community members within my community were talking behind our backs because my mom died of HIV/AIDS.’ (P1, 24 years old, Female, Pharmacist Assistance)

Children in her neighbourhood rejected her younger siblings, fearing that they might be carrying HIV/AIDS, she said:

‘Also, the kids that were mostly friends with my siblings, now it’s like, my siblings were discriminated against because of the HIV/AIDS that my mom had. I also feel like maybe they think even us, HIV/AIDS infected us [*looks worried*].’ (P1, 24 years old, Female, Pharmacist Assistance)

According to Levey et al. (2017), ‘the vast majority of children orphaned by AIDS or made vulnerable by HIV are ostracised, discriminated against, and isolated due to the shame associated with the disease’.

## Discussion

Youths who have experienced the untimely death of a family member should be helped to cope with their grief and have a positive outlook on their future.

Matthews (2017) says there are no linear patterns, ‘normal’ actions or formulae for grief. Death affects families emotionally, physically, spiritually and structurally. According to Lekalakala-Makgele (2018), each person’s sorrow is unique. Different types of grief expression, such as sobbing, fear and wrath, are universal; nonetheless, culture should be considered while appraising persons who have endured severe loss. Most bereaved can overcome their sadness, but sometimes it is extended or complex.

This article discussed difficult changes in the daily lives of the next of kin of the deceased. As youngsters were forced to become the breadwinners and leave school at a fairly young age, changes occurred. The loss of a family provider had a significant impact on young people and created difficulties in their lives. Their lack of self-management was caused by minimal help from family members who gave all of their own assistance to siblings.

The family had to survive and had financial requirements. Most of the people who passed away in this research were family breadwinners; therefore, their deaths left the household without any income. Some were forced to rely on government assistance for grants.

When a parent or senior relative passes away, the family is frequently left in confusion. The participants’ families in this research were at conflict with one another. These fights damage and hurt the young people involved. They also had no idea how to handle the challenges they encountered as well as the humiliating treatment they received as a result of conflicts. Therefore, a strategy must be created to assist the youth in managing their emotions, stop family issues and promote peace. The youngsters should foster a feeling of support within the family. By recognising their needs and encouraging them to go to a counsellor if they need to express their emotions and difficulties, youths should act as an advocate for their siblings.

Within communities, there is still stigma associated with HIV/AIDS. The effects of being ostracised by their own peers in the community are still felt by the children of individuals who died from HIV/AIDS. Youngsters in this research experienced discrimination at school and on the playgrounds. Youths had to deal with the stigma associated with HIV/AIDS and community members slandering them behind their backs.

The guidelines were developed based on the findings and results of the study. These guidelines are developed to assist nurses working in CHC on how to deal with or advise youth presenting with such problems or difficulties for consultation. The following actions address the Guideline.

### Youths should take ownership to lead and take up the responsibilities left behind by the deceased breadwinner to mentor and motivate the siblings and young ones

Taking ownership is doing something and accepting responsibility for it. Also, taking ownership means being responsible and making an active, passionate commitment (Golod 2018). Breadwinners are family providers. Today, a breadwinner might be either a man or a woman, or both (Kagan 2020).

These developed guidelines allow nurses to stay pleasant while teaching and supporting low-income teens. These guidelines also emphasise community activities to promote HIV/AIDS awareness and eliminate stigma.

### Changing the daily routines of the youths

Natural reward approach:

- The nurse should encourage youth to modify their habits and survive without loved ones. A family member’s death changes feelings, responsibilities and obligations (Johnson et al. 2016:126).

### Youths as role models

Natural reward approach:

- The nurse should explain role models to the kids. The nurse should advise youth to take it upon themselves to be role models in the family by exhibiting drive and optimism. Young family members look up to someone they know, according to Johnson et al. (2016:126). Young people in the family look up to someone they know personally.
- Nurses should advise and encourage youths to be able to explain what it means to follow a departed breadwinner.
- Nurses should advise and encourage youths to be able to explain what it is like to replace a departed breadwinner.
- Nurses should advise and encourage youths to identify their interests or abilities and discuss ways to get money from them.
- Nurse should encourage youth not to fear exploring new hobbies and share their life experiences and challenges with bereaved people.

Behavioural-focused approach:

- When youth visit healthcare facilities, the nurses should advise and encourage them to seek a government subsidy or establish a small company.
- Nurses should advise and encourage youths to be able to reduce their monthly expenses and learn more about a cost-effective health budget to save money.
- Nurses should advise and encourage youths to be a guide to their younger siblings by helping them to perform little duties at events or working weekend jobs to produce financial resources for the family.

### Being in emotional control

Cognitive behavioural approach:

- Nurses should advise and encourage youths to learn skills for managing their emotions, preventing family disagreements and bringing harmony to the home. Due to their inability to cope with life without the assistance of their loved ones, youths who have lost a family member suffer from anxiety and sadness. In youths, depression caused by traumatic events has a more significant detrimental impact on quality of life than posttraumatic stress disorder (Haravuori et al. 2016:46).
- Nurses should encourage youths to foster a friendly environment within their families and make room for everyone.
- Nurses should encourage youths to be advocates for their siblings by recognising their needs and proposing they see a counsellor for sorrow and challenges.
- Nurses should encourage youths to attend Department of Health community initiatives to combat the stigma associated with HIV/AIDS.
- Despite mortality, youth should hope for a better future. Nurses should advise and encourage youths to rebuild without the deceased family member since losing a loved one means being separated and losing the end as planned (Carlsson et al. 2020).

### Future research, limitations and strengths

This research in Cape Town, Western Cape, explored the experiences of youths who lost a family member to HIV/AIDS. The youngsters’ overall experiences following the death of a loved one were not pleasant. As a result of the findings, nurses can utilise the guidelines to educate teenagers on how to manage themselves after a family member has died of HIV/AIDS.

Nursing education recommendations: The care of young people who have experienced trauma should become part of the Bachelor of Nursing programme. Students need access to brief counselling courses as part of their mandatory curriculum (as required by the South African Nursing Council). Nurses may expand their expertise and advance their careers by reading up on the advantages of social work, counselling and youth self-management (Hlophe 2020:94).

Nurses should be allowed to learn about counselling and provide therapeutic sessions to bereaved individuals and families. It will provide them with the improvement of their understanding of dealing with grief and provide the most appropriate care for persons who are mourning (Hlophe 2020:94).

Nurses should instruct clinic visitors on grief steps. Most participants mourning a family member did not know where to seek help (Hlophe 2020:94).

Nursing research recommendations: The researcher suggests a qualitative study on the social effect of HIV/AIDS-related mortality on the young. The researcher also advises a male-only study. Males were missing from the clinic, indicating they were not seeking care for sorrow, sadness and perplexity.

All participants were women, although the inclusion criteria included males and females who had lost a family member to HIV/AIDS.

### Recommendations

If the young person is unable to cope with loss, a behavioural-focused approach should be taken. Youth should follow up with the CHC’s nurse to discuss referral to a clinical or pastoral psychologist. Children and youths should be kept under close observation if they are experiencing complex grieving because they may become trapped and unable to move through the mourning process on their own (Strachan-Proudfit 2019:1). In order to help the teenager through this difficult time, the nurse should give the teen space to talk about their fears and cry if they need too. It is crucial to ensure people to voice their opinions and feelings (Hlophe 2020:94).

As part of a natural reward system, the youth might show they are making an effort to improve their situation (by, for example, getting an education or a job) and recognise their worth to the family by taking responsibility for their actions. As part of stress management, adolescents should make a weekly plan to ensure they receive enough sleep, exercise, eat healthily, keep themselves engaged by connecting with positive people, attend church and find a job (Hlophe 2020:94). Supporting not just their ability to cope and survive, but also their incredible strength, development and resiliency will help them heal and flourish (Strachan-Proudfit 2019).

It is essential for young people to have a positive view. The cognitive-behavioural approach can be useful in two ways. Firstly, the young people should discuss the positive outlets for their enthusiasm, such as volunteer work. Volunteering at retirement communities is another option open to them (Hlophe 2020:94). Secondly, by opening a dialogue on HIV/AIDS, we may help young people understand that the disease is not related to factors such as age, socioeconomic status, religion, race or IQ, but is instead a part of our daily life (Hlophe 2020:94).

## Conclusion

The research limitation for this study was that only one CHC facility in Cape Town’s Khayelitsha urban region was studied. Only 18–25-year-olds who visited a CHC in Cape Town after losing a family member to HIV/AIDS were included in the research. The participants who were willing and partook in the study were women, although the inclusion criteria also aimed at including men.

Many people list the death of a parent or other close relative among the most traumatic events they have ever experienced. Others say the death was unnecessary and impossible to accept (Hlophe 2020:94). All too frequent reactions to the death of a loved one include feelings of shock and disbelief at the magnitude of the loss, as well as anguish and reluctance to fully accept the reality of the situation. The deceased was considered the family’s rock, and youth should understand that going through the phases of sorrow is normal and that there is no set period for each stage of grief – it depends on your relationship with the deceased (Hlophe 2020:94).

Regardless of the limitation, the study gave a great perspective of the experiences of youth following the death of a family member to HIV/AIDS. This study also developed guidelines for nurses to advise youths on self-management after losing a family member to HIV/AIDS.
